# Supplementary material for: Patterns of Intron Gain and Loss in Fungi
Source: PLoS Biol. 2004 Nov 30;2(12):e422. doi: 10.1371/journal.pbio.0020422 (PMC532390; doi:10.1371/journal.pbio.0020422)
Supplement: Table S1 — Also available at http://genes.mit.edu/NielsenEtAl/. (4.3 MB ZIP). [file pbio.0020422.st001.zip › NielsenEtAl/html/907.html]

AN8755.1.NCU02481.1.MG02616.1.FG00176.1


```
 CLUSTAL W (1.82) Multiple Sequence Alignments - Introns Inserted


Sequence 1: NCU02481.1	618 aa
Sequence 2: MG02616.1	607 aa
Sequence 3: FG00176.1	599 aa
Sequence 4: AN8755.1	613 aa
Alignment Length: 628 aa
Number Identitical Residues: 335 aa
Alignment Score (without introns) 15866


MG02616.1 	MLRTIPTRVLRRTSAAASRPASFCRCGR-LLSTTPRRAMPPITSTS---------LPSVN
NCU02481.1	MLRRAIPRISRRTVPAQTSSPLFGLCARPVRTSTTTSAHARLLSTSAARMTAPSPLAPVE
FG00176.1 	MMRRLSTRAPQRLISAASKS----------RSCAPLLAIRAISSTS--------VAKMSS
AN8755.1  	MLRSIPRRVPRRLPIFTTTATAGGPSR--LAQRAFTCGYLRMSPSS---------LPPVQ
          	*:*    *  :*     : ..    .       :   .   : .:*             .

MG02616.1 	PPLTAALPSDSFHLLPEAKKAGAAEDALYEQQVRDVEAWWATPRYAGITRPYTAADVVSA
NCU02481.1	HPFSSTIPFESFQLLPEEQKAGAAEDALLQQQIKEVEAWWASPRFKGIKRDWTAADVVSK
FG00176.1 	LSTHATQPADAFQLLPESQKPGEAEDRLYEATVHEIEAWWASPRFAGIKRPYSAEDVATK
AN8755.1  	PPVSSTLPSDSYQLLSTADKAGDAEDALYEQQIKDVEAWWNSPRFEGIKRPYSAADVVSK
          	 .  :: * ::::**.  .*.* *** * :  ::::**** :**: **.* ::* **.: 

MG02616.1 	RGSQQQSYPSSTMARKLWNLIQERKAEGKPIHTL~GAIDPIQMTQQAAHQEVLYVSGWAC
NCU02481.1	RGSLQQTYTSSVMARKLWNLVQERYAQGKPIHTL~GAIDPVQMTQQAPHQEVLYISGWAC
FG00176.1 	RGTQKVQYPSSVMAQKLFNLIREREAKGEPIHTM1GAIDPVQMTQQAPHQEVLYISGWAC
AN8755.1  	RGSLQQTYPSSLMARKLFNLLNERAAENKPVHTM1GAIDPVQMTQQAPNQEVLYVSGWAC
          	**: :  *.** **:**:**:.** *:.:*:**: *****:******.:*****:*****

MG02616.1 	SSVLTSTNEVSPDFGDYPYNTVPNQVQRLAKAQSMHDRKHWDARRKMSAQERSSTPYTDY
NCU02481.1	SSVLTSTNEVSPDFGDYPYNTVPNQVQRLHKAQSMHDRKQWFLRSKMTPEERAKTPYTDY
FG00176.1 	SSLLTSTNEVSPDFGDYPYNTVPNQVQRLAKAQNMHDRKQWDLRRKMTAEERTKTPYTDY
AN8755.1  	SSVLTTTNEVSPDFGDYPYNTVPNQVQRLFKAQQLHDRKHWDARRKMTPEQRKSTPYIDY
          	**:**:*********************** ***.:****:*  * **:.::* .*** **

MG02616.1 	LRPIIADGDTG~HGGLTAVTKLAKLFAEAGAAAVHFEDQMHGGKKCGHLAGKVLVSTGEH
NCU02481.1	FRPIIADGDTG~HGGLTAVMKLAKLFAENGAAGVHFEDQMHGGKKCGHLAGKVLVPTGEH
FG00176.1 	LRPIIADGDTG~HGGLSAVLKLAKLFAENGAAAVHFEDQLHGGKKCGHLAGKVLVPIGEH
AN8755.1  	MRPIIADGDTG2HGGLTAVLKLAKLFAENGAAAVHFEDQMHGGKKCGHLAGKVLVPIGEH
          	:********** ****:** ******** ***.******:***************. ***

MG02616.1 	INRLTAARMQWDIMGTENLVIARTDSESGRLISNNIDARDHEFILGVTDPSAAPLAGTLQ
NCU02481.1	INRLKAARFQWDVMGTENLVIARTDSESGKLLSSSIDARDHEFILGVADPSITSLAETLA
FG00176.1 	INRLVATRFQWDVMGCENLVIARTDSESGKLLSSAIDVRDHEFILGVADPSIEPLAETIQ
AN8755.1  	INRLVATRFQWDMMGVENLVIARTDSESGKLLSSAIDVRDHEFILGVTEES-EPLAETLQ
          	**** *:*:***:** *************:*:*. **.*********:: *  .** *: 

MG02616.1 	NMEARGASASEIDAYEAAFTRDHPLVTFDEA~AVSHMKKHNVDP---AEYEAGVAKDRDM
NCU02481.1	DMEARGAPGSEIDAYEAEWVKNTKLVTFDEA~ALAHFEHHNVRQDLFATYKATIAQNPNM
FG00176.1 	AMEAKGAAGAEIDAFEAQWVKNTKLITFDEA~AVAHMKKEGVEQSKIDEYLAETDKDHDM
AN8755.1  	AMEREGAAPSEIDAFELDWVKRHKLVTFDEA1VDAHLEAEGAPQAARDAYKKRVKENPDL
          	 ** .**. :****:*  :.:   *:***** . :*:: ...       *     :: ::

MG02616.1 	SIWDRRALAKDILGADKPDVYWDWDVPRTREGYYHFRSGMRAATKRALAFAPYADLLWVE
NCU02481.1	GITARRALAQSIT-PEHP-VYWDWDVPRTREGFYHFRNGMAAATKRALAFGPYADLLWVE
FG00176.1 	GISRRRGLANKYT--KEP-VYFNWDVPRTREGFYHYRAGMQAATKRALAFGPYADLLWVE
AN8755.1  	SITRRRELANDYT--KTP-VVWSCDIPRTREGFYHYRAGFPAATKRAKEFGPYADLLWVE
          	.*  ** **:.    . * * :. *:******:**:* *: ******  *.*********

MG02616.1 	TGDPSVSVCRQLGRAVKEAYPEKALVYNLSPSFNWMGHGFTEQTLKSFIWDIAKEG~FVL
NCU02481.1	TGDPSVEVATELGRAVREQFPGKGLVYNLSPSFNWMAHGFTQDTLKSFVWDIAKEG~FTL
FG00176.1 	TGDPNVQVASKLGRAVREVYPGKGLVYNLSPSFNWMAHGFTDETLKSFIWDIAREG~FVL
AN8755.1  	TGDPNVEKAAKLAGEVRAALPGKKLVYNLSPSFNWMGQGFDEASLKSFIWDLAQHG2FVL
          	****.*. . :*.  *:   * * ************.:** : :****:**:*:.* *.*

MG02616.1 	QLVSLAGVHTNATATCELARAFKDEGMLAYVNLVQRKEKEIGCDVLTHQKWSGAAYMDRI
NCU02481.1	QLVSLAGLHSTATISNELARRYKNEGMQAYVELVQKREKELGVDVLTHQKWSGANYVDAI
FG00176.1 	QLISLAGLHSNATITTELSREFKKDGMKAYVDIVQRREKDLKCDVLTHQKWSGASYIDGI
AN8755.1  	QLISLAGLHSGATITAELSRAFKDEGMLAYVRLIQAREKELGVDVLTHQKWSGAPYMDGI
          	**:****:*: ** : **:* :*.:** *** ::* :**::  *********** *:* *

MG02616.1 	VGAIQSGSSTSKSMGAGNTEG-~-----QFH--
NCU02481.1	LGHIQSGSSSSKSMGEGNTEN-~-----QFV--
FG00176.1 	LGAIQSGSSSSKSMGEGNTEG-~-----QFH--
AN8755.1  	VGAIQSGSSSSKSMGEGNTEKD1SEWGTEIHER
          	:* ******:***** **** . :. .::: .
```
